# Analysis of Behavioural Characteristics Related to Unintentional Injury in Southeast Chinese Adolescents: Evidence from a School-Based Survey

**DOI:** 10.3390/ijerph14030241

**Published:** 2017-03-01

**Authors:** Wei Feng, Qinghai Gong, Kui Liu, Hui Li

**Affiliations:** 1 Department of Chronic Diseases and Community Health, Fenghua District Center for Disease Control and Prevention, Ningbo 315010, Zhejiang Province, China; fhfwhhxx1111@163.com; 2 Department of Chronic Disease Prevention, Ningbo Municipal Center for Disease Control and Prevention, Ningbo 315010, Zhejiang Province, China; gongqinghai@163.com; 3 Department of Science Research and Information Management, Zhejiang Provincial Center for Disease Control and Prevention, Hangzhou 310051, Zhejiang Province, China; kliucdc@yeah.net

**Keywords:** unintentional injury, influence factors, students, logistic regression analysis

## Abstract

The purpose of this study was to explore the epidemiological features of common unintentional injury-related behaviours and to identify possible factors that lead to these unsafe behaviours among adolescents. A representative sample of 10,806 students was recruited from 77 schools by using the two-stage stratified random sampling method. All participants took a self-administered questionnaires and data were analysed to estimate the prevalence of unintentional injury-related behaviours and to identify the influential factors for these behaviours. The prevalence of unsafe swimming, jaywalking, illegal bicycling and not wearing a seat belt was 6.35%, 33.08%, 18.10% and 15.73%, respectively. The proportion of students who had two, three or four unintentional injury-related behaviours was 14.59%, 4.27% and 0.57%, respectively. Multiple regression analysis showed that male adolescents, living in an urban area and attending a vocational-technical school might contribute to the occurrence of four unintentional injury-related behaviours. In addition, the marital status of parents and father with a college degree or above were negatively associated with the adolescent’s behaviour of not wearing a seat belt. Considering diverse epidemiological characteristics of unintentional injury-related behaviours among adolescents, targeted interventions such as enhancing self-protection capabilities and strengthening safety consciousness by family, school and related departments should be implemented to lower the occurrence of unintentional injury-related behaviours.

## 1. Introduction

Injury were one of the most common causes of death in children and adolescents around the world [1,2,3]. In China, injury was ranked as the leading cause of accidental death of about 50,000 cases among the 0–14 age range [4]. Unintentional injury, as the most common component of injury, included drowning, accidental falls, road traffic injuries, poisoning and burns [1,5]. According to reports of the world health organization, the most common unintentional injuries were road traffic injuries, falls, burns, drowning and poisoning [2]. Previous studies determined that the rate of unintentional injury was from 11.34% to 13.86% and that the rate was higher in rural regions than in urban regions [6,7,8]. Additionally, unintentional injury in adolescents accounted for 60% of all deaths [9].

According to the Chinese comprehensive survey report of health-related risk behaviours in adolescents, we defined unintentional injury-related behaviour as some common risk behaviours could directly cause or activate the occurrence of unintentional injury in daily life [10]. To our knowledge, there was a close relationship between unintentional injury-related behaviours and unintentional injury. However, due to the variations of the economic level and living environment in different countries, potential risk behaviours were also diverse. Thus, investigations of possible risk behaviours would be discrepant in different countries. Combined with available literature, this research chose the common risk behaviours that led to unintentional injury in China, including unsafe swimming, jaywalking, illegal bicycling and not wearing seat-belts [10]. Considering the large sample size of our survey, it had the merit to recognise the true risk behaviours in the adolescent population, particularly in China.

In this study, we performed a cross-sectional survey to identify the epidemic pattern of unintentional injury-related behaviours in adolescents in Ningbo Municipality, China, and analysed the potential influential factors attributed to these unsafe behaviours, which all could contribute to providing a scientific basis for developing an effective intervention strategy.

## 2. Materials and Methods

### 2.1. Study Design and Sampling Method

This cross-sectional investigation was carried out from October to December in 2015 and covered 11 regions that included five districts and six counties in Ningbo city. The respondents were students (grades 7–12) enrolled in middle school, senior high school and vocational-technical school in Ningbo city. A two-phase stratified cluster random sampling method was used to select the participants, and self-administered questionnaires were applied to investigate unintentional injury-related behaviours. The first phase initially extracted all schools classified as middle school, senior high school and vocational-technical school in 11 regions in Ningbo city. All of these schools were sorted by geographic region in north to south orientation and west to east orientation. After that, three middle schools, two senior high schools and two vocational-technical schools were randomly selected in each county or district. The second phase extracted the classes from the included schools. Classes in the same school were sequenced from junior to senior by class number. Then, a simple random sampling method was applied to randomly select eight classes in each middle school, six in senior high school and six in vocational-technical school. Finally, there were 24 classes from middle school, 12 classes from senior high school and 12 classes from vocational-technical school in each district or county. All students from the selected classes participated in the questionnaire survey. Eventually, 10,806 students from the selected classes participated in the questionnaire survey, and 10,790 valid questionnaires were used to analyse the characteristics of unintentional injury-related behaviours.

### 2.2. Ethics Statement

All participants were informed of the research purpose and informed consent was obtained from each participant before carrying out the research. This study was approved by the Ethics Committee of the Ningbo Municipal Center for Disease Control and Prevention (No. 201503).

### 2.3. Personnel Training and Questionnaire Survey

The self-administered questionnaire survey was anonymous. Local Centres for Disease Control and Prevention initiated the unified training and organised the implementation of this study. Specifically, all respondents were convened in their classroom simultaneously and the questionnaires were completed independently without a school teacher being present. The researchers were onsite when the survey was conducted. Survey contents included basic characteristics and information regarding unsafe swimming, jaywalking, illegal bicycling and not wearing a seat belt.

### 2.4. Indicators

Unsafe swimming referred to having swam without safety measures in the past 12 months. Jaywalking referred to having run a red light in the past 12 months. Illegal bicycling referred to the behaviour of running a red light, taking over a vehicular road, riding double, turning without a gesture or retrogradation by bicycle in the past 12 months. Not wearing a seat belt was defined as not having worn a seat belt while sitting in the front seat of a car in the previous month.

### 2.5. Statistical Analysis

All data were recorded using EpiData 3.0 software (Odense, Denmark) by two individuals. A descriptive analysis was carried out to illustrate the distribution of characteristics among participants. A multiple logistic regression model was used to determine potential influential factors for unintentional injury-related behaviours. Ultimate results in tables were displayed as an odds ratio (OR) with its 95% confidence interval (95% CI) correspondingly. All available data were analysed by SPSS Version 13.0 (SPSS Inc., Chicago, IL, USA). A two-sided *p*-value < 0.05 was considered as statistically significant.

## 3. Results

### 3.1. General Characteristics of All Participants

We had recruited 10,806 students, of which 10,790 (99.85%) completed the questionnaires. There were 5485 male respondents and 5305 female respondents with a sex ratio of 1.03:1. Of all included respondents, 5824 respondents were from urban areas and the rest were from rural areas. According to the school types, 5272 were from middle school, 2948 from senior high school and 2570 from vocational-technical school. The details were shown in Table 1.

### 3.2. Analysis of Single Unintentional Injury-Related Behaviours

According to the definition of each indicator, the proportion of four unintentional injury-related behaviours involving unsafe swimming, jaywalking, illegal bicycling and not wearing a seat belt among respondents was 6.35%, 33.08%, 18.10% and 15.73%, respectively. Concerning the distinct gender, the proportion of unsafe swimming, jaywalking and illegal bicycling was significantly higher in male than in female. With regard to different area, the prevalence of not wearing a seat belt was lower in urban areas contrasted to rural areas, whereas the prevalence of the other three behaviours was significantly higher in urban areas compared to rural areas. In various school types, adolescents attending vocational-technical school tended to have the highest prevalence of all unintentional injury-related behaviours. These information was displayed in Table 2.

### 3.3. Analysis of Combined Unintentional Injury-Related Behaviours

All respondents with two potential risk behaviours accounted for 14.59%, of which 4.27% had three potential behaviours and 0.57% had four potential behaviours (Table 3). Besides, the results suggested that the proportion of two combined behaviours was higher in male than in female, and were higher in urban areas than in rural areas. Regarding the diverse school types, students who attended vocational-technical school had the highest prevalence of two combined behaviours while those attending middle school had the lowest prevalence. Furthermore, findings from three combined related behaviours analysis were similar to the results of two combined behaviours analysis. However, a four combined behaviours analysis only identified the difference in gender that males had a higher prevalence.

### 3.4. Analysis of the Potential Factors That Influenced Unintentional Injury-Related Behaviours

In this study, multiple regression analysis was performed to identify the possible influential factors attributed to unintentional injury-related behaviours. The finding showed that male adolescents, living in an urban area and attending a vocational-technical school might contribute to the occurrence of four unintentional injury-related behaviours. Besides, married status of parents and father with a college degree or above were negatively associated with the adolescent’s behaviour of not wearing a seat belt. These details were shown in Table 4.

## 4. Discussion

To the best of our knowledge, due to the discrepancies of economic level, living environment, study population and lifestyle in different countries, studies for the probable unintentional injury-related behaviours were inconsistent. For instance, in America and Thailand, unintentional injury-related behaviours commonly involved whether a bicycle helmet was worn or not, a seat belt was worn or not, driving when drunk or driving with other irrelevant behaviours [11,12]. In China, unintentional injury-related behaviours generally included unsafe swimming, jaywalking, illegal bicycling and not wearing a seat belt [10,13,14,15].

In this study, we found that of the four evaluated behaviours, jaywalking had the highest proportion in Ningbo (33.08%), followed by illegal bicycling, which were similar to previous studies conducted in Yinchuan City, Yuxi City and Shandong province [13,14,15]. With advances and development of social economics, this phenomenon could be explained by the sharp increase in the number of motor vehicles but without relevant improvements in other aspects such as related traffic safety education, suggesting that lack of traffic knowledge and weak safety awareness were still existed in student population. Besides, walking and bicycling in China were still the main trip mode for the student population. Thus, strengthening traffic safety education was an urgent need in juvenile groups.

Although available literature reported that the proportion of drowning deaths in rural regions was significantly higher than in urban regions, our study revealed a lower proportion of unsafe swimming in rural regions than in urban regions [16,17]. Yet it was not a contradiction for the results above. Due to poor protective implements and limited resources in rural areas, the occurrence of drowning was more common in these areas. Thus, the government and local schools tended to focus on drowning safety education that resulted in a decline in the prevalence of unsafe swimming, which was also proved in a study conducted in the Pudong area of Shanghai City [18].

Another interesting finding of our study was the gender difference in unintentional injury-related behaviours. The occurrence of unsafe swimming, jaywalking and illegal bicycling was significantly higher in schoolboys than in schoolgirls. Moreover, schoolboys also showed a higher prevalence of combined unintentional injury-related behaviours. Compared to schoolgirls, the odds ratio and 95% CI of three and four combined unintentional injury-related behaviours were 3.08 (2.48–3.81) and 8.95 (3.85–20.79), respectively. This might be associated with the boys’ natural behaviour. Therefore, more concerns should be highlighted on schoolboys in these aspects. Additionally, vocational-technical school tended to have the highest prevalence for four unintentional injury-related behaviours, which might be caused by weaker safety education. Thus, more courses about safety education should be considered in vocational-technical school.

Previous studies indicated that parents with high educational level could reduce the child’s injury rate more obviously [19,20,21]. In our study, multiple logistic regression analysis only showed that father with a college degree or above was negatively correlated with the behaviour of not wearing a seat belt. This outcome might be explained by the phenomena that in China most drivers in the family were male. That is to say, a father with higher educational level might have higher traffic safety consciousness, being a role model for their children.

However, our study had some limitations: (1) Given we did not collect data of unintentional injury from respondents, the association between unintentional injury-related behaviour and potential injury was not further analysed; (2) Partial indicators collected from the past year might cause an unavoidable recall bias in our design; (3) Other potential factors including hiding some unsafe behaviours and disturbances from other classmates might also influence the prevalence of risk behaviours in our study; (4) Considering the limited respondents with two or three unintentional injury-related behaviours simultaneously, we did not conduct the analysis in different combinations; (5) In this study, we only performed our research in one city, the representativeness of which could be influenced by some specific factors as economics and education level. Therefore, some potential bias might disturb the extrapolation of our conclusions.

## 5. Conclusions

Overall, considering the diverse epidemiological characteristics of unintentional injury-related behaviours among adolescents, targeted interventions such as enhancing self-protection capabilities and strengthening safety consciousness in the adolescent population, should be implemented by family, school and other related departments to effectively lower the occurrence of unintentional injury-related behaviours.

## Figures and Tables

**Table 1:** General characteristics of the study participants.

Variable	Participants	Proportion (%)
Gender		
Male	5485	50.83
Female	5305	49.17
Region		
Urban	5824	53.98
Rural	4966	46.02
School Type		
Middle School	5272	48.86
Senior High School	2948	27.32
Vocational-technical School	2570	23.82
Marital Status of Parents *		
Married	9859	91.37
Divorce or Other	931	8.63
One Child or Not		
Yes	6945	64.37
No	3845	35.63
Educational Level for Father		
Primary School or Below	1888	17.50
Junior-senior High School	6956	64.47
College Degree or Above	1946	18.04
Educational Level for Mother		
Primary School or Below	2572	23.84
Junior-senior High School	6462	59.89
College Degree or Above	1756	16.27
Live Status ^#^		
Parents	8810	81.65
Parent	752	6.97
Others	1228	11.38

* Marital Status of Parents included the status of married, divorced or other (widowed, separated or unavailable); ^#^ Live status referred to the specific guardian who the respondent lived with. It included parents, parent and others (grandparents, maternal grandparents and other relatives).

**Table 2:** Single behaviour characteristics of unintentional injury in the student population.

Variable	Unsafe Swimming	OR and 95% CI	Jaywalking	OR and 95% CI	Illegal Bicycling	OR and 95% CI	Not Wearing Seat-Belt	OR and 95% CI
Gender								
Male	506 (9.23%)	2.91 (2.44–3.47)	2033 (37.06%)	1.45 (1.33–1.57)	1416 (25.82%)	2.78 (2.50–3.08)	877 (15.99%)	1.04 (0.94–1.15)
Female	179 (3.37%)	1	1536 (28.95%)	1	591 (11.14%)	1	820 (15.46%)	1
Region								
Urban	419 (7.20%)	1.37 (1.17–1.61)	2338 (40.14%)	2.04 (1.87–2.21)	1256 (21.57%)	1.54 (1.40–1.71)	760 (13.05%)	0.65 (0.58–0.72)
Rural	266 (5.36%)	1	1231 (24.79%)	1	751 (15.12%)	1	937 (18.87%)	1
School Type								
Vocational-technical School	199 (7.74%)	1.31 (1.09–1.57)	1200 (46.69%)	2.19 (1.98–2.41)	523 (20.35%)	1.21 (1.07–1.36)	552 (21.48%)	2.08 (1.83–2.35)
Senior High School	168 (5.70%)	0.94 (0.78–1.14)	862 (29.24%)	1.03 (0.94–1.14)	563 (19.10%)	1.12 (0.99–1.25)	531 (18.01%)	1.67 (1.47–1.89)
Middle School	318 (6.03%)	1	1507 (28.58%)	1	921 (17.47%)	1	614 (11.65%)	1

**Table 3:** Combined behaviours characteristics of unintentional injury in the student population.

Variable	Two Combined Related Behaviours	OR and 95% CI	Three Combined Related Behaviours	OR and 95% CI	Four Combined Related Behaviours	OR and 95% CI
Gender						
Male	970 (17.68%)	1.67 (1.50–1.87)	347 (6.33%)	3.08 (2.48–3.81)	55 (1.00%)	8.95 (3.85–20.79)
Female	604 (11.39%)	1	114 (2.15%)	1	6 (0.11%)	1
Region						
Urban	985 (16.91%)	1.51 (1.36–1.69)	297 (5.10%)	1.57 (1.30–1.91)	32 (0.55%)	0.94 (0.57–1.56)
Rural	589 (11.86%)	1	164 (3.30%)	1	29 (0.58%)	1
School type						
Vocational-technical School	492 (19.14%)	1.60 (1.41–1.82)	178 (6.93%)	2.25 (1.81–2.79)	15 (0.58%)	0.99 (0.54–1.84)
Senior High School	404 (13.57%)	1.08 (0.94–1.23)	114 (3.87%)	1.22 (0.95–1.55)	15 (0.51%)	0.87 (0.47–1.60)
Middle School	678 (12.86%)	1	169 (3.21%)	1	31 (0.59%)	1

**Table 4:** Identification of the possible influential factors leading to the four unintentional injury-related behaviours by multiple logistic regression analysis.

Influence Factors	Unsafe Swimming	Jaywalking	Illegal Bicycling	Not Wearing a Seat Belt
	OR (95% CI)	OR (95% CI)	OR (95% CI)	OR (95% CI)
Gender				
Male	2.92 (2.44–3.49)	1.44 (1.32–1.56)	2.77 (2.49–3.09)	1.20 (1.08–1.34)
Female	1	1	1	1
Region				
Urban area	1.21 (1.03–1.43)	2.04 (1.87–2.23)	1.39 (1.25–1.54)	0.69 (0.62–0.77)
Rural area	1	1	1	1
School type				
Vocational-technical School	1.44 (1.19–1.73)	2.32 (2.09–2.57)	1.37 (1.21–1.55)	1.98 (1.74–2.25)
Senior High School	1.05 (0.86–1.28)	1.16 (1.04–1.28)	1.25 (1.11–1.41)	1.72 (1.51–1.96)
Middle School	1	1	1	1
The only child or not				
Yes	1.04 (0.88–1.28)	0.99 (0.90–1.08)	0.96 (0.86–1.07)	0.95 (0.84–1.06)
No	1	1	1	1
Marital Status				
Marriage	0.74 (0.54–1.01)	0.84(0.71–1.00)	0.82 (0.67–1.01)	0.71 (0.58–0.88)
Divorce or others	1	1	1	1
Educational Level for Father				
College degree or above	0.89 (0.65–1.23)	0.94 (0.79–1.11)	0.91 (0.75–1.11)	0.63 (0.50–0.79)
Middle school	1.02 (0.82–1.27)	1.01 (0.90–1.14)	0.89 (0.77–1.02)	0.91 (0.79–1.04)
primary school	1	1	1	1
Educational Level for Mother				
College degree or above	0.83 (0.60–1.13)	0.83 (0.71–0.98)	1.02 (0.84–1.24)	0.80 (0.64–1.01)
Middle school	0.89 (0.73–1.09)	0.95 (0.85–1.01)	1.04 (0.91–1.18)	0.99 (0.88–1.13)
Primary school	1	1	1	1
Live Status of adolescents				
Parents	0.93 (0.73–1.20)	0.88 (0.77–1.01)	0.99 (0.84–1.16)	0.82 (0.70–0.97)
Single parent	0.86 (0.59–1.26)	0.92 (0.75–1.13)	0.99 (0.77–1.26)	0.88 (0.68–1.12)
Others	1	1	1	1
